# Relationship of Two Vertical Jumping Tests to Sprint and Change of Direction Speed among Male and Female Collegiate Soccer Players

**DOI:** 10.3390/sports4010011

**Published:** 2016-02-16

**Authors:** Isaiah T. McFarland, J. Jay Dawes, Craig L. Elder, Robert G. Lockie

**Affiliations:** 1 Department of Health Sciences, University of Colorado-Colorado Springs, 1420 Austin Bluffs Blvd, Colorado Springs, CO 80923, USA; imcfarland@uccs.edu (I.T.M.); celder@uccs.edu (C.L.E.); 2 Department of Kinesiology, California State University, Northridge, 18111 Nordhoff St, Northridge, CA 91330-8272, USA; robert.lockie@csun.edu

**Keywords:** soccer, agility, power, change of direction speed, linear speed

## Abstract

In collegiate level soccer acceleration, maximal velocity and agility are essential for successful performance. Power production is believed to provide a foundation for these speed qualities. The purpose of this study was to determine the relationship of change of direction speed, acceleration, and maximal velocity to both the counter movement jump (CMJ) and squat jump (SJ) in collegiate soccer players. Thirty-six NCAA Division II soccer players (20 males and 16 females) were tested for speed over 10 and 30 m, CODS (T-test, pro agility) and power (CMJ, SJ). Independent t-tests (*p* ≤ 0.05) were used to derive gender differences, and Pearson’s correlations (*p* ≤ 0.05) calculated relationships between the different power and speed tests. Female subjects displayed moderate-to-strong correlations between 30 m, pro agility and T-test with the CMJ (*r* = −0.502 to −0.751), and SJ (*r* = −0.502 to −0.681). Moderate correlations between 10 and 30 m with CMJ (*r* = −0.476 and −0.570) and SJ (*r* = −0.443 and −0.553, respectively) were observed for males. Moderate to strong relationships exist between speed and power attributes in both male and female collegiate soccer players, especially between CMJ and maximal velocity. Improving stretch shortening cycle (SSC) utilization may contribute to enhanced sport-specific speed.

## 1. Introduction

The ability to start, stop and change directions rapidly and efficiently is essential for success in most team sports [1,2,3,4]. This is especially true for sports that require intermittent repeat sprints, such as soccer. Within a 90 min soccer match, players may perform over 600 changes of direction [5] and numerous linear sprints ranging in distance between 1.5 and 105 m. These high-intensity speed bouts are critical to the game’s outcome and ultimately a team’s success [2,6,7].

There appears to be a significant relationship between speed and one’s ability exert force rapidly [8]. Subsequently, the countermovement vertical jump (CMJ) and squat jump (SJ) are two commonly used field tests to assess lower-body power [9]. Numerous studies indicate that a significant relationship exists between sprint acceleration performance and SJ height. Similarly, the CMJ has been shown to demonstrate moderately strong correlations to maximal sprint velocity [10,11].

Several studies have also revealed an association between measures of leg power and change of direction speed (CODS) [12,13,14,15]; however, these results have been inconsistent. In a study conducted by Fatih (2009) [12], moderate relationships between vertical jump performance and CODS (Hexagonal Obstacle Test) were discovered among both adolescent boys (*p* < 0.05) and girls (*p* < 0.01). Barnes *et al.* (2007) [13] found the CMJ was a significant predictor of agility performance (34% variance) among female collegiate volleyball players in which the athletes performed four 5 m sprints with three 180° turns. Lockie *et al.* (2014) [16] investigated the relationship between unilateral jump performance and multi-directional speed in male team-sport athletes (*n* = 30). Left leg vertical jump (LVJ) had no notable correlations with T-test scores, however right leg vertical jump (RVJ) did (*r* = −0.380 to −0.512, *p* ≤ 0.05). Left-leg standing broad-jump (SBJ) and lateral jump (LJ) correlated with both the T-test and 505 agility tests (*r* = −0.370 to −0.729, *p* ≤ 0.05). This demonstrates a relationship between CODS and power in male athletes as well, however the existence of such relationships seem to be dependent on the CODS test being performed, as well as whether a jump test is conducted bilaterally or unilaterally. These varied results may be due to the diversity in both study populations and different CODS tests performed.

It is also important to determine whether there are gender differences in lower-body power measured by jump performance, and linear speed and CODS. Possible physiological and biomechanical differences that may influence the strength of the relationship between power production and speed qualities include muscular strength, technique, motor abilities, and anatomical difference (*i.e.*, Q-angle, femoral notch width) [17,18]. The relationships between leg power, as measured by the SJ and CMJ, and linear speed and CODS requires further investigation in collegiate soccer players, both from a performance and gender perspective.

Therefore, the purpose for this study is to investigate the production of power as measured by the SJ and CMJ and their relationship to the performance of acceleration, maximal velocity, and CODS among NCAA Division 2 male and female soccer players [9]. Please note that as SJ and CMJ are not direct measures of power, jump height is a measurable result of power production that is highly applicable to performance in many sports, and serves as an easy, cost effective field assessment for strength and sport coaches. Sport and strength coaches could design efficient and relevant skill testing/training regimens to better improve performance qualities by incorporating power tests that are more strongly associated with acceleration, maximal velocity and CODS as independent speed attributes.

## 2. Materials and Methods

### 2.1. Subjects

Performance testing data for thirty-six (Male = 20, Female = 16) NCAA Division II soccer players was used for this analysis. All subjects were between 18 and 23 years of age, a mean height of 67.71 ± 3.50 cm for males and 64.21 ± 2.68 cm for females, and mean weight of 75.80 ± 7.73 kg for males and 62.37 ± 7.07 kg for females. Institutional Review Board approval was obtained to conduct data analysis on this archival data set. Nonetheless, the study still conformed to the recommendations of the Declaration of Helsinki. All testing was performed indoors on a hardwood basketball court in single session.

### 2.2. Procedures

Two different pre-season testing sessions were performed in close proximity, one for males and one for females. Athletes were requested to wear clothing that would not restrict movement (*i.e.*, shorts, sweats, and athletic wear) and to wear a good pair of athletic shoes. All testing was conducted indoors on a hardwood basketball court in order to maintain a consistent testing surface and eliminate extraneous variables, such as wind or rain that may confound testing results. After anthropometric information was measured and recorded for each athlete, a standardized warm-up, led by a certified strength and conditioning specialist, was conducted prior to testing. This warm-up lasted approximately 10 min and consisted of light walking, jogging, and dynamic stretching. Once the warm-up was completed the testing began. All tests were performed on the same day under the direction of the university’s athletic performance staff. Testing order was arranged in a manner to ensure that one test would interfere with the performance of another test. Subjects also received an oral and visual demonstration of the proper techniques required to successfully complete each test before they were asked to perform them. Subjects were then allowed up to three sub-maximal trials to practice technique. Once the submaximal trials were completed the athletes performed each assessment. The SJ and CMJ were performed at the same time in a randomized fashion. Immediately following these measures the athletes were randomly assigned to either perform the pro-agility or T-test. The order of both agility tests were randomized in order to reduce any order effects, as well as maximize the allotted amount of time to conduct the testing session. Once all CODS tests were completed the athletes performed the 30 m sprint.

### 2.3. Anthropometric Data

Anthropometric information, including height (cm) and weight (kg) measurements were collected using standard procedures on a doctors beam scale (Cardinal; Detecto Scale Co, Webb City, MO, USA).

### 2.4. Squat Jump and Countermovement Jump Test

Vertical jump height for the SJ and CMJ was measured using an electronic jump mat (Just Jump, ProBotics Inc., Huntsville, AL, USA). This device calculates vertical jump height by measuring flight time against gravity (9.81 m/s), and when compared to the jump and reach Vertec measuring device the Just Jump Mat system has been shown to be a valid method (*R* = 0.906) for measuring vertical jump height [19]. All athletes were instructed to step on the mat, place their hands on their hips, and when ready, jump as high as possible. For the SJ the athletes were further instructed to lower themselves to the bottom of a jumping position so that an angle of approximately 90° knee flexion was achieved, hold for a minimum of 2 s, and proceeding through a strictly concentric jumping motion. Each athlete performed the SJ and CMJ in a randomized order, recording the best of 3 attempts for each. The Sayers power equation (Peak power (Watts) = 60.7 × jump height (cm) + 45.3 × body mass (kg)—2055) was used to estimate total power output in watts. This information was normalized for each athlete by dividing power output by total body mass to determine the power to weight ratio (P:W) for SJ and CMJ.

### 2.5. Pro-Agility Shuttle

Athletes stood on the starting line and on the “go” command sprinted 5 yards to the right, touch the designated line with the right foot, immediately sprinted 10 yards to the left and touched the designated line with the left foot, then completed the shuttle by sprinting through the start line. Similar to previous research, a hand held stopwatch was used to record the time required to complete this test, with times rounded to the nearest 0.10 s, and the best of three trials was recorded [20].

### 2.6. T-Test

The T-test used the protocol outlined by Paulo *et al.* [21]. Time required to complete each trial was measured with a Speed Trap II (TC-System, Brower Timing Systems, Draper, UT, USA) automatic timing device. This device was set up on the starting line. To perform the test athletes assumed a staggered stance just behind the starting line. The timer started when the crossed the infrared beam and stopped when they crossed the beam a second time upon returning to the start line. The best of three trials was recorded, and rounded to the nearest 0.10 of a second.

### 2.7. Thirty Meter Sprint Test

Sprint speed (10 m and 30 m) were measured using an electronic timing system (TC-System, Brower Timing Systems, Draper, UT, USA). The 10 m time provided a measure of acceleration, while 30 m time measured maximal velocity sprinting. Each athlete was allowed three attempts with the best time being recorded to the nearest 0.10 s. using a staggered stance, athletes positioned themselves behind the starting line with their front foot on a weight sensitive timing pad. The timer started when the athlete stepped the pad, and stopped after sprinting the full 30 m distance. Timing gates were set at both the 10 and 30 m marks in order to measure both acceleration and maximal velocity. The best of three trials was recorded, and rounded to the nearest 0.10 of a second.

### 2.8. Statistical Analysis

Collected data was entered into a computer file suitable for statistical analysis using the Statistics Package for Social Sciences (Version 23.0; IBM Corporation, New York, NY, USA). A descriptive statistical analysis was conducted to determine the mean scores and standard deviations for the total sample on all anthropometric and performance scores. Between groups comparisons were analyzed using a series of independent *t*-tests. Pearson’s correlations were also performed to determine the relationships between all four speed and CODS measures, and the squat jump and countermovement jump tests. Relationships between all speed and CODs and estimated peak power in watts, in relative an absolute terms were investigated (*p* = 0.05). In addition, significance level was set at *p* ≤ 0.05 for all calculations and r-values were interpreted as weak (≤0.39), moderate (≤0.40–0.69) or strong (≥0.70) [22].

## 3. Results

Descriptive statistics for both groups are displayed in Table 1. When separated by gender, female collegiate soccer players displayed strong correlations between (PAPw) and CODS, as well as the 30 m sprint (Table 2). Male players displayed moderate correlations between PAPw and both the 10 and 30 m sprints, but not CODS. For the females, the 30 m was almost as strongly related to CMJ as SJ, and CMJ displayed considerably stronger relationships to the pro-agility shuttle and T-test than did SJ. In male soccer players, a slightly stronger correlation was seen between CMJ and both the 10 and 30 m compared to the SJ. The power to weight (P:W) ratios calculated for both the CMJ and SJ displayed moderate to strong correlations with the various speed qualities, however they were inconsistent across the different tests and gender.

**Table 1:** Descriptive statistics.

Performance Test	Gender (M = 20; F = 16)	Mean ± SD
SJ (cm)	F	40.15 ± 4.66
	M	54.81 ± 6.70
CMJ (cm)	F	41.85 ± 4.98
	M	58.47 ± 6.53
10 m	F	1.92 ± 0.31
	M	1.63 ± 0.12
30 m	F	4.78 ± 0.22
	M	4.16 ± 0.14
Pro Agility (s)	F	5.36 ± 0.18
	M	4.64 ± 0.25
T-test (s)	F	11.920 ± 0.56
	M	10.22 ± 0.41
PAPw (CMJ)	F	3310.54 ± 474.11
	M	4927.71 ± 567.16
P:W (CMJ)	F	53.08 ± 5.03
	M	86.13 ± 5.17
PAPw (SJ)	F	3207.43 ± 448.91
	M	4705.703 ± 616.91
P:W (SJ)	F	51.44 ± 4.60
	M	62.10 ± 4.96

Notes: SJ = squat jump; CMJ = counter movement jump; PAPw = peak anaerobic power in watts; P:W = power to watt ratio.

**Table 2:** Correlations (r-values) of selected measures of power with acceleration, maximal speed and CODS by gender.

Performance Test	10 m (Acceleration)		30 m (Max Speed)		Pro Agility		T-Test	
	M	F	M	F	M	F	M	F
SJ	−0.44	−0.31	−0.55 *	−0.68 *	−0.32	−0.50 *	−0.23	−0.68 *
CMJ	−0.47 *	−0.22	−0.57 *	−0.67 *	−0.30	−0.58	−0.16	−0.76 *
PAPw (CMJ)	−0.49 *	−0.53 *	−0.48 *	−0.53 *	0.03	−0.50 *	0.02	−0.46
P:W (CMJ)	−0.30	−0.10	−0.45 *	−0.63 *	−0.45 *	−0.60 *	−0.25	−0.79 *
PAPw (SJ)	−0.43	−0.61 *	−0.44	−0.53 *	0.01	−0.44	−0.02	−0.41
P:W (SJ)	−0.32	−0.23	−0.48 *	−0.65 *	−0.48 *	−0.53 *	−0.34	−0.72 *

Notes: SJ = squat jump; CMJ = counter movement jump; PAPw = peak anaerobic power in watts; P:W = power to watt ratio; *: *p* ≤ 0.05.

## 4. Discussion

In the sport of soccer, power has been linked to the ability to accelerate, achieve maximal velocity and effectiveness in COD tasks [1,2,3,4,10,11,12,13,14,15]. By definition, acceleration refers to the capacity to rapidly increase speed [10]. As such, starting strength, or the ability to rapidly generate working force, is a major factor [23]. This elicits the functional significance of lower-body power in the production of acceleration. Compared to the CMJ, slightly stronger correlations have been found between SJ and acceleration over distances of 0–20 m (*r* = −0.43 to −0.76, respectively) [12,13,14]. This was further reinforced by our findings with female soccer players, though the correlation between CMJ was only marginally lower (*r* = −0.317 SJ, *r* = −0.222 CMJ, *p* ≤ 0.05). However, for the males SJ had a slightly lower correlation with acceleration compared to CMJ (*r* = −0.443 SJ, −0.476 CMJ *p* ≤ 0.05). Results showing stronger correlations between SJ and acceleration may be due to the fact that adequate starting strength is necessary to initiate acceleration and overcome inertia and does not rely as heavily on the SSC due to a static starting position [24]. However, in the sport of soccer, players are often already in motion when a sprint is initiated [2]. In this case, the SSC is already contributing to force generation during a moving or rolling start. This means that mechanically it is more similar to a maximal sprint where short ground contact time and the assistance of stored elastic energy as seen in the CMJ is now a contributing factor. Given this knowledge, it may be more beneficial for coaches to focus on assessment tests and exercise selections, which improve the SSC utilization when working with collegiate soccer players. It should be considered that other variances in acceleration performance outcomes including maximal leg strength and technique are unaccounted for and may have a strong influence on such performance outcomes [25].

Strong correlations were shown between maximal velocity and power output in both male and female soccer players in this study. For males, the CMJ had a slightly stronger correlation when compared to SJ. For the females the opposite was seen, with SJ showing slightly stronger correlations with maximal velocity than CMJ. This is in agreement with previous research studies [12,13]. This further indicates the possible benefits of maximal velocity obtained from improved utilization of the SSC.

When considering gender differences the test results and the training and assessment indications vary for males and females. Female soccer players demonstrated that the CMJ was more strongly correlated with CODS (*r* = −0.58 Pro Agility, *r* = −0.76 T-test, *p* ≤ 0.05). In the males, we observed only minor relationships between CMJ and CODS (*p* > 0.05). This is similar to the results seen by Barnes *et al.* (2007). Barnes *et al*. (2007) discovered strong correlations between CMJ and CODS utilizing short sprints and 180° turns in female athletes. Furthermore, there appears to be relatively consistent findings showing CMJ as a good predictor of CODS performance in females [10]. It is difficult to determine if the observed differences are due to gender or the specific tests used in the research studies. For example, a contributing factor could be the volume of linear sprinting that exists within a COD test. COD assessments that feature more linear sprinting may reduce the influence of COD ability, and rely more heavily on characteristics important for linear speed, such as the SSC qualities of muscles [26,27]. Future research into the relationship between leg power, and a measure such as exit velocity from a COD, would be beneficial [27,28,29,30].

It may be of note that the authors of this paper decided to focus on the SJ and CMJ heights achieved due to their practical applications. The results of this study may only be applicable to division II collegiate soccer players specifically. Other weaknesses of this study include the sample size being limited to division II varsity soccer teams for only one university, as well as the exclusion of other power tests such as weighted jumps squats or drop jumps. Other such tests could be indicative of other power categories such as reactive strength, and their impact on the development of the various speed qualities pertinent to successful performance in soccer.

## 5. Conclusions

The results of this study indicate moderate to strong relationships between speed and power attributes in both male and female collegiate soccer players, especially between CMJ and maximal velocity. By including exercises that improve SSC utilization when training soccer players, improved development of acceleration, CODS, and especially that of maximal velocity may occur. If time restraints during testing sessions are also an issue, as they often are, the coach may choose to only use CMJ for power testing as it seems to have stronger correlations with respect to all of the above speed attributes pertinent for soccer performance. It is unclear as to whether or not the results of this study would apply to all soccer populations as our study sample consisted of male and female division II collegiate soccer players. Future studies observing different populations and competitive levels within the soccer community will further enhance our knowledge regarding the relationships between power production and various speed performance. It may also be advantageous to investigate power production differences and their relationships between different field positions (*i.e.*, defenders, midfielders, forwards and goalkeepers). Also, further investigation into the correlations witnessed between both P:W and PAPw may contribute to the understanding of physiological and performance relationships between power production and speed qualities in field sport athletes. Collectively, this knowledge will further benefit sport specific training and player development.
